# ASCO's Leadership Development Program: Focusing on the Next Generation of Leaders in Asia Pacific

**DOI:** 10.1200/GO.22.00313

**Published:** 2023-02-22

**Authors:** Roselle B. de Guzman, Melvin L.K. Chua, David Goldstein, Soe Aung, Vanessa Eaton, Keunchil Park

**Affiliations:** ^1^ Manila Central University-FDT Medical Foundation Hospital, Caloocan City, Philippines; ^2^ Department of Head and Neck and Thoracic Cancers, Division of Radiation Oncology, National Cancer Centre Singapore, Singapore; ^3^ Nelune Cancer Centre, Prince of Wales Hospital, Randwick, Australia; ^4^ Myanmar Oncology Society, University of Medicine 1, Yangon, Myanmar; ^5^ American Society of Clinical Oncology, Alexandria, VA; ^6^ Samsung Medical Center, Seoul, South Korea

## Abstract

The demand for high-quality and more comprehensive oncology care will continue to rise globally. Effective leadership is particularly important.

**METHODS:**

The evolving endeavor of ASCO to reach out globally has covered the development of the next-generation leaders from Asia Pacific. Through the Leadership Development Program, the untapped talent of the region and the future leaders in oncology will gain the knowledge and skill sets, which prepares them to navigate the complex dynamics of oncology health care.

**RESULTS:**

The region is the largest and the most populous with more than 60% of the world's population. It has 50% of cancer cases and is estimated to account for 58% of cancer deaths worldwide. The demand for high-quality and more comprehensive oncology care will continue to rise in the years to come. This growth will intensify the need for capable leaders. Leadership styles and behaviors are different. These are shaped within the context of cultural and philosophical views and beliefs. The pan-Asian interdisciplinary group of young leaders are expected to gain knowledge and skillsets through the Leadership Development Program. They will learn to work on strategic projects within a team and gain knowledge about advocacy. Communication and presentation skills and conflict management are also important components of the program. Through learning culturally relevant skills, participants can effectively collaborate with others, build relationships, and lead within their own institutions and societies and within ASCO.

**CONCLUSION:**

Institutions and organizations need to have a deeper and more sustained focus on leadership development. Successfully addressing the challenges on leadership development in Asia Pacific is important.

## INTRODUCTION

Developing new leaders is critical for any organization. The complexity of health care organizations and the pressing challenges of modern health care call for great leadership from within, thus providing the motivation to develop physician leaders.^1-6^ Traditionally, the criteria set to advance physicians to leadership positions are based on academic and clinical accomplishments. Good clinicians and researchers do not necessarily make good and effective leaders. Distinct competencies for leadership in medicine are different from the competencies and skills needed in the practice of the profession.^7-9^ There should be a balance between managerial skills focused on efficiency, financial stringency, and human resource optimization and skills focused on patient care.^10^ Consequently, the clinical and scientific skills must form part of the managerial focus and are able to be provided if there is an identified management role for physicians.^11,12^ It is unknown to what extent previous medical training and experiences of doctors affect the performance of physician executives. Previous studies support the importance of including doctors in hospital governing boards.^13-19^ Given the complexity encountered within modern health care organizations, it is difficult to demonstrate the impact of leadership on outcomes when assessing medical and nonmedical leadership in the same setting. It remains unclear in the literature whether nonfaculty career administrators or clinical staff are better suited.^20,21^ Studies found no difference in the performance of medical and nonmedical managers.^22-25^

**CONTEXT**

**Key Objective**
How important is it to build and develop the next generation of oncology leaders in the Asia Pacific region?
**Knowledge Generated**
ASCO through its Asia Pacific Regional Council has initiated the Leadership Development Program to create a pool of oncology professionals from the region. Building effective Asian leaders with diverse backgrounds, culture and specializations will bridge the leadership gap and build leadership capacity in Asia, the region with the heaviest cancer burden.
**Relevance**
The complexity of modern health care and the demands in the field of oncology call for empowered, competent, and motivated leaders to help improve the quality of cancer care in Asia Pacific.

Identifying the fundamental principles of physician leadership is critical.^26^ The principles are the core components that characterize leadership competency, and these abilities are teachable skills.^27-29^ Physician leadership has been recognized to correlate with organizational success.^30^ Implementing a physician leadership program cultivates and strengthens the skills of emerging clinical leaders and develops a pipeline of physician leaders.^31,32^ Oncology is facing enormous challenges and changes. Despite much progress in cancer management, the increasing number of cases and mortality globally^33^ is a continuous challenge. This calls for strong leadership from cancer care providers and adequate succession planning to ensure continuity of purpose.

ASCO is committed to promoting lifelong learning and professional development of oncology professionals. In 2009, ASCO launched its Leadership Development Program (LDP)^34^ where participants learn strategies and critical skillsets, gain exposure to the roles and mission of ASCO, and network with and receive mentorship from ASCO leaders. In 2013, eligibility for the prestigious LDP was extended to international members. Three participants from Asia had been admitted to this program.^35^ Although there are different challenges for leaders in countries outside the United States, the LDP provided the participants a strong foundation of leadership skills with impactful and long-lasting personal and professional benefits.^35^

## BRIDGING THE LEADERSHIP GAP AND BUILDING LEADERSHIP CAPACITY IN ONCOLOGY IN ASIA PACIFIC

The Asia Pacific (APAC) region is composed of a diverse group of high-, middle-, and low-income countries. Each has distinctive cultural and socioeconomic backgrounds, which pose unique challenges in delivering high-quality, equitable patient care.^36-38^ Asia has the heaviest cancer burden because of its high population density comprising 60% of the global population. The region has 50% of cancer cases and is estimated to account for 58% of cancer deaths worldwide.^39^

In an effort to learn more about these challenges and understand the landscape of oncology in APAC, ASCO launched the Asia Pacific Regional Council in May 2019. The Council, representing the eastern APAC countries, advises ASCO on the needs of members in the region and facilitates and encourages member involvement in the society's global activities.^40^ The Council recognized the need to develop leaders from the region and to further provide members with a new avenue to contribute to ASCO's work.

The demand for a high-quality and comprehensive cancer care highlights the challenges and opportunities in developing oncology leaders in APAC. In a study performed by the Center for Creative Leadership,^41^ in the top 200 organizations (by revenue), leaders of Asian ethnicity and/or nationality represent only about 4% of the executive teams in US-headquartered companies and 3% in Europe-headquartered firms. It is unknown if similar data would suggest very limited representation of ethnic Asian talent in oncology leadership roles. One explanation on the under-representation of Asians in leadership positions in the United States is an issue of cultural fit. There is a mismatch between East Asian's low assertiveness in communication and the American norms of how leaders should communicate.^42^

The Council has been tasked with adapting content and lessons learned from the LDP to offer a new leadership program tailored to the needs of an APAC audience. The first-ever ASCO APAC LDP was thus launched in Nov 2021.^43^ The program was funded by the Conquer Cancer, the ASCO Foundation Mission Endowment.

## A HISTORICAL AND CULTURAL CONTEXT OF LEADERSHIP IN THE REGION

With more than half of the world's population, the region's role in the global socioeconomic landscape and in global health is becoming increasingly important. Workplaces are becoming more multicultural. Organizations, companies, and institutions are expanding globally. Asian leaders should be ready to navigate these challenges ahead. There are common demands that leaders face across the world. However, leadership styles and behaviors are different and are shaped within the context of cultural and philosophical views, beliefs, and approaches.

Traditionally, Asians have a strong respect for age and seniority. They are conditioned to revere seniors, follow established customs and accepted norms, and avoid opposing viewpoints. Filial piety, an attitude of respect for parents, elders, and ancestors in societies, is influenced by Confucian thought. It has shaped family care giving and other aspects of individual, social, political, and legal relations in Asian countries. Filial attitudes emphasize obedience. It has been related to resistance to cognitive change, which tends to adopt a passive, uncreative, and uncritical orientation toward learning.^44,45^ Therefore, these attitudes tend to result in loyalty and groupthink and may hamper creative thinking and innovation.

The characters of different Asian leadership styles are also reflected not only from the influences of Confucianism but also from Daoism, Mohism, and other philosophy.^46-48^ Leadership is built on a foundation of moral character and exercised through virtuous examples. A leader or a ruler is a moral manager and a person of benevolence, wisdom, and courage.^46,49^ A Daoist leader needs to be trustworthy and unbiased and use action over words. Such leadership should promote harmony and balance.^50^ The Mohism philosophy and major ethical tenets promote universal, unbiased respect and concern for all regardless of affiliations or relations.^48^

Some Asian countries were under colonization and gained freedom from foreign political rule. Most earned their independence through protests and resistance or after going through the ravages of war. Asian leaders had to put much work on changing institutional structures, policies, and laws, which are rooted in the colonial past. There are individuals who may not be used to having the freedom to find and develop their own leadership strengths and style. Some are not accustomed to developing themselves to their full potential. These are historical roots born from decades and even centuries of colonial oppression.

Asian countries have inherently humble cultures with strong family and societal bonds. A paternalistic leadership style is built on the premise that dad knows best. This is claimed to be the one dominant leadership style in Asia.^51^ Several research studies have looked into the elements of paternalistic leadership.^52-55^ It entails authoritarianism, benevolence, and moral character.^56^ The moral character dimension of paternalistic leadership expects the leader to behave on high moral standards, and the subordinate sees them as a role model, believes in their moral integrity and benevolence, and follows their authoritarian guidance.

Authoritarian leadership is characterized by the hierarchical dynamics between the leader's authority, control, and power. It is also marked by the compliance, obedience, and respect of the subordinates. Benevolent leaders provide individualized and holistic concern to the subordinate. A recent meta-analysis found that two dimensions of paternalistic leadership, benevolent leadership and moral leadership, were positively associated with employee innovation. By contrast, the dimension of authoritarian leadership was negatively associated with innovation.^56^

There are strategies that stakeholders must adopt to maximize leadership talent in the region. Focusing on strength-based leadership culture will develop leaders with authentic and unique strengths.^57^ A strong culture of coaching and mentoring can give some flexibility to the rigid and hierarchical Asian culture. Giving recognition for significant and unique contributions is a powerful motivator for emerging leaders. Building cultural intelligence^58^ is particularly important as individuals exposed to working in different environments and cultural contexts adapt to a diverse range of scenarios and situations.

## SHAPING THE APAC LDP AND DEFINING THE INTENDED AND TANGIBLE OUTCOMES

The objectives of the LDP are thus to create a pool of oncology professionals from APAC who are empowered to identify and fill leadership roles in oncology, to learn the skills for strategic planning tools and time management that will prepare them for potential future advocacy roles, and to promote interdisciplinary collaboration across the region. The pan-Asian interdisciplinary group of young leaders are expected to gain knowledge and skillsets including

1. Understanding the different types of leaders and how to apply individual strengths to be an effective leader.
2. Knowledge about advocacy, importance of advocacy efforts, and ability to participate in advocacy initiatives.
3. Ability to collaborate with others on regional efforts to improve cancer care.
4. Presenting information to others and delivering media interviews.
5. Effectively managing conflict and ability to have difficult conversations.

After the yearlong program, key performance indicators on the success and the impact of the program will look at

1. Collaborative projects fostered between participants (joint regional educational initiatives; regional cancer research, health services research, etc).
2. Appointments to various leadership positions within ASCO, national committees, and regional and international societies and committees.

After graduating from the program, participants are able to stay connected to ASCO through opportunities to serve as volunteers in advisory groups, committees, and working groups. They could be in roles where they excel and where their talents are truly leveraged. As they develop senior leadership roles within their own countries and the region, they can also serve as ambassadors between their institutions or local societies and ASCO, organizing or participating in networking events and collaborative initiatives that would keep the engagement and make it a strong and well-connected community.

Since the program represents diverse countries, resource settings, gender, and specialties, participants will learn shared views on leadership models from the different Asian countries. The participants will gain knowledge on the key differences that are unique to specific Asian countries. This knowledge will stand them in good stead in formulating culture- and resource-appropriate strategies and guidelines for cancer control activities. The program would also highlight crucial factors for a leader in Asia to succeed, which may not be familiar to faculties from the West.

## PROGRAM INITIATION AND DEVELOPMENT—CHALLENGES AND LESSONS LEARNT

### Profiling the APAC Leader in Oncology

The selection criteria had to be modified given the diverse backgrounds of the candidates who would come from various countries. However, some of the overarching key criteria for assessment of leadership qualities were retained from the original program, which included leadership roles in their departments, civic engagement, and clinical or research groups; strong track records of new project initiatives in their institutions; active involvement in national society committees; and a strong letter of support from their current mentors and heads of departments.

Collectively, the Council intended to ensuring diversity at all levels, be it country, sex, and seniority with the candidates' selection. English language competency was a necessity, given that the program will be conducted in English. Another thing considered during the profiling of the candidates was their disparate environments, which correlated with their country's income status (as defined by the World Bank classification).^59^ It was apparent that leaders from institutions of developed countries had more resources to kickstart programs. In the same vein, the leaders from different countries faced divergent sets of challenges; for example, leaders from low- and middle-income countries were restrained by a chronic lack of resources and manpower,^60-62^ whereas leaders from more developed ecosystems were more vocal about institutional bureaucracy. Nonetheless, ASCO and the Council were open to exploring how the leaders from such diverse backgrounds and environments would interact overall and in their small project groups.

### Selecting the Pioneer Batch of APAC Young Oncology Leaders

As this was the pioneer cohort, there were very limited insights into the background and caliber of the applicants. To encourage applications, publicity was done via several streams—word of mouth by the council members, through the national societies, through electronic dissemination by e-mails to the ASCO international membership, or through social media platforms. In total, there were 45 applicants from 12 different countries, and impressively, the applicants were well-matched in terms of their clinical and leadership track records. This was a testament to the appeal and enthusiasm toward an ASCO-led LDP in APAC.

Selection was led primarily by the Council members, who were unanimous in ensuring diversity for the pioneer class. The members prioritized equal opportunities during the selection process, so that candidates were not penalized because of limitations imposed on them by the country that they represent. A challenge that was, however, encountered was the difficulty in organizing interviews for the large number of applicants by the Council. This inevitably resulted in a process that was heavily dependent on the sentiments of the Council to the candidates' curricula vitae and personal statements, leading to some degree of uncertainty surrounding the suitability of the chosen candidates. Going forward, the Council has opined on the need to review this process and to consider the inclusion of a face-to-face interview either in-person or online.

### Conducting the LDP in a Virtual World Amidst a COVID-19 Pandemic

Apart from the candidate selection process, the other big unknown for ASCO and the Council were the feasibility of conducting an online leadership course and whether such a platform is conducive for coaching and mentoring and learning for the leaders. Unlike the US LDP, which consisted of four quarterly sessions of 2-3 days of intensive boot camps, the APAC LDP was split into 12 monthly plenary sessions conducted exclusively online over Zoom. Each plenary session was participated by all the 12 leaders (Table 1), along with three mentors, three coaches, two course directors, and ASCO education staff who oversaw the curriculum and conduct of the LDP.

**TABLE 1:**
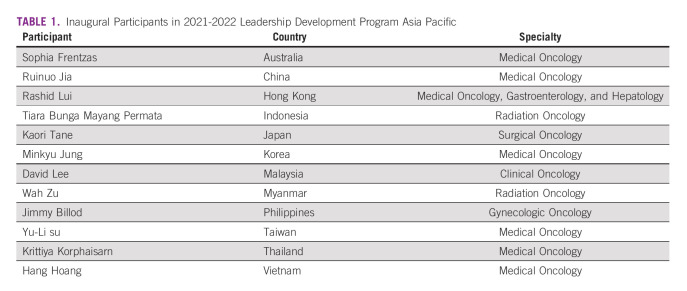
Inaugural Participants in 2021-2022 Leadership Development Program Asia Pacific

At the initial phase of the program, it was evident that an online forum was not ideal to foster interaction between the leaders themselves and with the faculty members. This lack of participation is compounded by the discrepancy in their abilities to communicate in English, which speaks to another blind spot of the candidate interview and screening process—something to be reviewed with the subsequent iterations of the APAC LDP. Nonetheless, participation levels gradually improved over time when the leaders, coaches, and mentors were given the opportunity to share about themselves before every plenary session, which helped to reduce the virtual social barriers.

Another inevitable challenge encountered by the leaders, coaches, and mentors is related to the stress and burden of having to undergo an intensive LDP course, while managing their routine clinical and administrative duties during the peaks of the COVID-19 pandemic. At times, it was obvious that some of the leaders were struggling to cope, and this affected the progress of respective small group projects (Table 2). Nonetheless, ASCO staff, coaches/mentors, and fellow team-mates were ready to aid and cover for each other to ensure that the projects were able to continue. All things considered, the first version of ASCO APAC LDP was a successful effort to provide training of the future leaders from this part of the world.

**TABLE 2:**
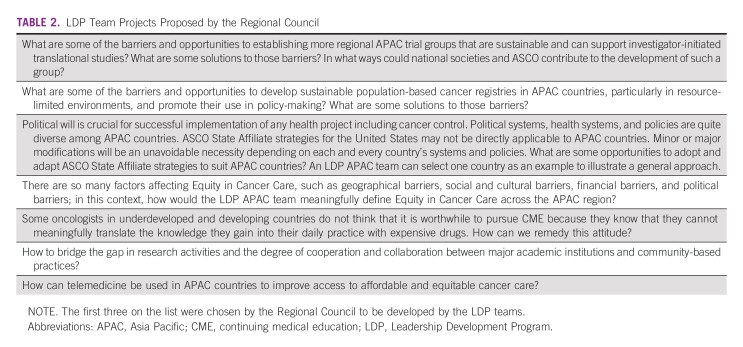
LDP Team Projects Proposed by the Regional Council

## BUILDING TOMORROW'S ASIAN LEADERS THROUGH PROJECT-BASED LEARNING

A key component of any leadership course is developing teamwork and cooperation. A pathway to achieve this is through the commissioning of a project for a team to develop and present a finalized proposal. Each project team was selected to ensure diversity of countries, specialties, and sex. The projects (Table 2) were put forth by various members of the Council and felt to be relevant to the regions' needs and the background of the selected candidates. Eventually, three projects were finalized on the basis of the highest number of votes by the Council.

Fundamental principles in project selection included a clear recognition that the region was diverse in socioeconomic and cultural aspects, that projects had the potential to make an impact on all countries by addressing problems common to all patients with cancer irrespective of where they lived, and that project outcomes incorporated flexibility to suit local needs and had clear tangible outcomes.

Each team consisted of one coach, one mentor, and four LDP participants who rotated serving as a team leader. The role of the coach was to provide information that team members requested, educate the team about the project topic, and provide networking leads and connections in support of the team's work on the project. The mentor provided guidance to focus and organize their work; monitored team progress against their plan with regular formal and informal assessments; and facilitated team communication, planning, decision making, conflict resolution, and lessons learned. Teams were encouraged to engage with other LDP teams and mentors.

Project-based learning was designed to encourage development of many skills including open communication, respect others’ views, encourage and build on all ideas, identify problems and barriers and explore solutions, and manage effective team meetings. Working on team projects allowed the participants to take ownership and responsibility, by developing models of teamwork and accountability. Team leadership was intended to nurture soft skills related to team management, such as facilitating the team members to define and implement their mission, goals, priorities, roles and accountabilities, operating protocols, and development of collaborative and mutually supportive relationships. Additional skills to be developed included ensuring that the team operates efficiently and that members participate actively in discussions and decision making; facilitating debates but also mitigating conflicts, clarifying issues to be addressed and keeping members focused; stakeholder engagement; and monitoring of members' performance against their assigned tasks, ensuring that they are meeting targets, promoting visibility and recognition of team member contributions and performance.

The projects are outcomes-driven, they represent goals that the Council wishes to promote to ensure better care of our patients and better engagement of health services throughout the region in key aspects of cancer treatment, prevention, and control. Equally, the projects are a vehicle to instill leadership qualities through experiential learning and mentoring.

## EASTERN AND WESTERN APPROACHES TO LEADERSHIP

Leadership has different styles, perspectives, and philosophical approaches in different parts of the world. Organizational structures of leadership also have some differences.^63-65^ Western organizations usually have flatter structures and are less prescriptive. Eastern organizations have hierarchical structures and therefore are more directive.^66^ Western leaders build open relationships. Superiors and subordinates perceive each other as inherently equal in hierarchy at work. This may mean that work organization, roles, and positions can be changed easily. On the other hand, Asian leaders maintain a distance with the subordinates affecting work organization, structure, and relations between them. Subordinates can have inhibitions in approaching their managers and superiors.^67^

Individualistic culture emphasizes the unique personal characteristics of an individual. The needs and motives are the focal point of understanding the individual's actions.^68^ Western approach to leadership tends to focus on individual achievement. Collectivistic culture places more emphasis on the identification of an individual within a group, such as roles and duties associated with being a group member. Eastern leadership approach focuses on the collective activities of followers.^69^ The ties between members of the group are very strong, and loyalty to the group is one of the basic values. Asian leaders focus on collective activities of followers and collective achievement. The traditional Western style of leadership may not be well suited to an Asian context, and given the high cancer burden, a fit-for-purpose model is essential.

Notably, as cancer care globalizes, modern approaches to leadership in the East and the West continue to evolve. Leaders should adopt principles from each other and effectively apply them in any culture, country, or region.

## SHAPING THE FUTURE OF LEADERSHIP IN ONCOLOGY IN APAC

APAC is the largest and most populous region with more than 60% of the world's population. There are more than 50 countries with diverse ethnic groups, histories, languages, cultures, environments, economics, and sociopolitical systems. Unfortunately, it has long been under-represented, despite the highest cancer burden, and suffers from a huge unmet need for optimal oncologic care.

Effective leadership is particularly important in difficult times. The best way to prepare future leaders in oncology is to get as diverse a set of experiences as possible under their belt. To prepare them to meet today's demands, stakeholders should support their development as a journey of change and growth. Leadership development is not only a yearlong program but also a career-long process. It requires continuous learning with multiple touch points, follow-ups, and accountabilities. The sense of purpose and mission is important to be reinforced. Young leaders are greatly energized and motivated when they are connected to a broader and higher purpose.

Institutions and organizations will benefit from a deeper and more sustained focus on leadership development. It is important to focus on the changes that are needed from the perspectives of leaders, rather than investing in showcase programs that may not address the immediate needs of the respective health care systems. Hence, successfully addressing the challenges on leadership development is important as we need a pipeline of future leaders. Organizations must ensure that their talented staff are constantly upskilling, getting ready for the challenges of tomorrow. They must remain committed to developing the skillsets and leadership qualities of the next-generation Asian leaders. These approaches lay the foundation for superior transformation and performance.

In conclusion, the demand for high-quality and more comprehensive oncology care will continue to rise across APAC in the years to come. This growth will intensify the need for capable leaders who are able to navigate the complex dynamics of health care services in the countries. The need for an Asian-focused leadership program was exemplified by the wide geographic spread of the participants. Critical capabilities such as collaboration and resilience will be must-haves to prepare the young leaders for whatever future Asia throws at them.

The evolving endeavor of ASCO to reach out globally will not only help discover the untapped talent of the region but through them improve the quality of cancer care in APAC. In addition to developing future leaders of the region, it will also provide the potential opportunity for rising APAC stars to serve in leadership positions for ASCO in due course. The Council is very proud to be involved in this ambitious program and confident that this will represent a great milestone for the whole APAC oncology society to move forward for quality care and global leadership of oncology.

## References

[1] Straus SE, Soobiah C, Levinson W: The impact of leadership training programs on physicians in academic medical centers: A systematic review. Acad Med 88:710-723, 2013 23524921 10.1097/ACM.0b013e31828af493

[2] Chadi N: Medical leadership: Doctors at the helm of change. Mcgill J Med 12:52-57, 2009 19753289 PMC2687916

[3] Chen TY: Medical leadership: An important and required competency for medical students. Tzu Chi Med J 30:66-70, 2018 10.4103/tcmj.tcmj_26_18 PMC5968745 29875585

[4] Leadership in health care; what defines a leader? https://www.usa.edu/blog/leadership-in-healthcare/

[5] Al-Sawai A: Leadership of healthcare professionals: Where do we stand? Oman Med J 28:285-287, 2013 23904925 10.5001/omj.2013.79 PMC3725246

[6] Lyons O, George R, Galante JR, et al: Evidence-based medical leadership development: A systematic review. BMJ Leader 5:206-213, 2021 10.1136/leader-2020-000360 37850339

[7] Stoller JK: Developing physician-leaders: A call to action. J Gen Intern Med 24:876-878, 2009 19455370 10.1007/s11606-009-1007-8 PMC2695517

[8] Warren OJ, Carnall R: Medical leadership: Why it’s important, what is required, and how we develop it. Postgrad Med J 87:27-32, 2011 20935344 10.1136/pgmj.2009.093807

[9] Stoller JK: Leadership essentials for CHEST medicine professionals models, attributes, and styles. Chest 159:1147-1154, 2021 32956716 10.1016/j.chest.2020.09.095 PMC7501065

[10] Berghout MA, Fabbricotti IN, Buljac-Samardžić M, et al: Medical leaders or masters? A systematic review of medical leadership in hospital settings. PLoS One 12:e0184522, 2017 28910335 10.1371/journal.pone.0184522 PMC5598981

[11] Savage M, Savage C, Brommels M, et al: Medical leadership: Boon or barrier to organisational performance? A thematic synthesis of the literature. BMJ Open 10:e035542, 2020 10.1136/bmjopen-2019-035542 PMC7375428 32699130

[12] Martinussen PE, Davidsen T: ‘Professional-supportive’ versus ‘economic-operational’ management: The relationship between leadership style and hospital physicians' organisational climate. BMC Health Serv Res 21:825, 2021 34399744 10.1186/s12913-021-06760-2 PMC8369705

[13] Prybil LD: Size, composition, and culture of high-performing hospital boards. Am J Med Qual 21:224-229, 2006 16849778 10.1177/1062860606289628

[14] Jiang JH, Lockee C, Bass K, et al: Board oversight of quality: Any differences in process of care and mortality? J Healthc Manag 54:15-29, 2009 19227851

[15] Veronesi G, Kirkpatrick I, Vallascas F: Clinicians on the board: What difference does it make? Soc Sci Med 77:147-155, 2013 23232025 10.1016/j.socscimed.2012.11.019

[16] De Andrade Costa L: The effect of physician board membership on uncompensated care provision. Appl Econ 46:2290-2300, 2014

[17] Bai G, Krishnan R: Do hospitals without physicians on the board deliver lower quality of care? Am J Med Qual 30:58-65, 2015 24413657 10.1177/1062860613516668

[18] Kuntz L, Pulm J, Wittland M: Hospital ownership, decisions on supervisory board characteristics, and financial performance. Health Care Manage Rev 41:165-176, 2016 25978002 10.1097/HMR.0000000000000066

[19] Sarto F, Veronesi G: Clinical leadership and hospital performance: Assessing the evidence base. BMC Health Serv Res 16:169, 2016 (suppl 2) 27230873 10.1186/s12913-016-1395-5 PMC4896259

[20] Andersson T: The medical leadership challenge in healthcare is an identity challenge. Leadersh Health Serv 28:83-99, 2015 10.1108/LHS-04-2014-0032 25921315

[21] Rothman DJ, Blumenthal D, Thibault GE: Medical professionalism in an organizational age: Challenges and opportunities. Health Aff 39:108-114, 2020 10.1377/hlthaff.2019.00186 31905069

[22] Schneller ES, Greenwald HP, Richardson ML, et al: The physician executive: Role in the adaptation of American medicine. Health Care Manage Rev 22:90-96, 1997 9143905 10.1097/00004010-199704000-00010

[23] Schultz FC, Pal S, Swan DA: Who should lead a healthcare organization: MDs or MBAs? J Healthc Manag 49:103-116, 2004 15074119

[24] Clay-Williams R, Braithwaite J: Doctors in Executive Management: A Systematic Review of the Peer-Reviewed Literature. Sydney, Australia, Centre for Clinical Governance Research, Australian Institute of Health Innovation, University of New South Wales, 2012

[25] Clay-Williams R, Ludlow K, Testa L, et al: Medical leadership, a systematic narrative review: Do hospitals and healthcare organisations perform better when led by doctors? BMJ Open 7:e014474, 2017 10.1136/bmjopen-2016-014474 PMC5623455 28947438

[26] Collins-Nakai R: Leadership in medicine. McGill J Med 9:68-73, 2006 19529813 PMC2687901

[27] Moore JM, Wininger DA, Martin B: Leadership for all: An internal medicine residency leadership development program. J Grad Med Educ 8:587-591, 2016 27777672 10.4300/JGME-D-15-00615.1 PMC5058594

[28] Berghout MA, Fabbricotti IN, Buljac-Samardzic M, et al: Medical leaders or masters? A systematic review of medical leadership in hospital settings. PLoS One 12:e0184522, 2017 28910335 10.1371/journal.pone.0184522 PMC5598981

[29] Lucas R, Goldman EF, Scott AR, et al: Leadership development programs at academic health centers: Results of a national survey. Acad Med 93:229-236, 2018 28658016 10.1097/ACM.0000000000001813

[30] Goodall AH: Physician-leaders and hospital performance: Is there an association? Social Sci Med 73:535-539, 2011 10.1016/j.socscimed.2011.06.025 21802184

[31] Berriochoa C, Amarnath S, Berry D, et al: Physician leadership development: A pilot program for radiation oncology residents. Int J Radiat Oncol Biol Phys 102:254-256, 2018 30191858 10.1016/j.ijrobp.2018.05.073

[32] Frich JC, Brewster AL, Cherlin EJ, et al: Leadership development programs for physicians: A systematic review. J Gen Intern Med 30:656-674, 2015 25527339 10.1007/s11606-014-3141-1 PMC4395611

[33] World Health Organization International Agency for Research on Cancer: GLOBOCAN 2020. https://gco.iarc.fr/today/home

[34] American Society of Clinical Oncology Leadership Development Program. https://www.asco.org/career-development/leadership-development-program

[35] De Guzman R, Malik M, Lopes GdL, et al: ASCO leadership development program: International perspectives. J Glob Oncol 4:1-3, 2018 10.1200/JGO.18.00014 PMC6223475 30241270

[36] Bhoo-Pathy N, Kong YC, Bustamam R, et al: Needs of cancer patients in an Asian setting. Ann Oncol 30:ix141-ix150, 2019

[37] Wilson BE, Jacob S, Yap ML, et al: Estimates of global chemotherapy demands and corresponding physician workforce requirements for 2018 and 2040: A population-based study. Lancet Oncol 20:769-780, 2019 31078462 10.1016/S1470-2045(19)30163-9

[38] Wells JC, Sharma S, Del Paggio JC, et al: An analysis of contemporary oncology randomized clinical trials from low/middle-income vs high-income countries. JAMA Oncol 7:379-385, 2021 33507236 10.1001/jamaoncol.2020.7478 PMC7844695

[39] Sung H, Ferlay J, Siegel RL, et al: Global cancer statistics 2020: GLOBOCAN estimates of incidence and mortality worldwide for 36 cancers in 185 countries. CA Cancer J Clin 71:209-249, 2021 33538338 10.3322/caac.21660

[40] American Society of Clinical Oncology: ASCO in Action Regional Council for a Stronger Society: ASCO's New Asia Pacific Regional Council Finishes Active First Year, 2020. https://www.asco.org/news-initiatives/policy-news-analysis/regional-council-stronger-society-ascos-new-asia-pacific

[41] Mallis E, Murray P: Global Asian Leader from Asia, for the World GAL 2.0 Partner. Asia Pacific: Center for Creative Leadership (CCL) Research, 2022. https://www.ccl.org/articles/research-reports/the-global-asian-leader-from-asia-for-the-world/

[42] Lu JG, Nisbett RE, Morris MW: Why East Asians but not South Asians are underrepresented in leadership positions in the United States. Proc Natl Acad Sci U S A 117:4590-4600, 2020 32071227 10.1073/pnas.1918896117 PMC7060666

[43] ASCO Connection: ASCO Congratulates Inaugural Participants in 2021‐2022 Leadership Development Program in the Asia Pacific Region 2021. https://connection.asco.org/magazine/asco-international/asco-congratulates-inaugural-participants-2021-2022-leadership

[44] Kwan K-LK: Counseling Chinese peoples: Perspectives of filial piety. Asian J Couns 7:1, 2000

[45] Ho DY: Filial piety, authoritarian moralism, and cognitive conservatism in Chinese societies. Genet Soc Gen Psychol Monogr 120:349-365, 1994. 7926697

[46] Schenck A, Waddey M: Examining the impact of Confucian values on leadership preferences. J Organ Educ Leadersh 3:4-7, 2017.

[47] Lee YT, Han A, Byron TK, et al: Daoist Leadership: Theory and Application. Cambridge: Cambridge University Press, 2010. https://www.cambridge.org/core/books/abs/leadership-and-management-in-china/daoist-leadership-theory-and-application/C70689B1E54A8F1F9FCD3D970A41440D#

[48] Fraser C: Mohism, in Zalta EN (ed): The Stanford Encyclopedia of Philosophy, Stanford, CA, Stanford University, 2022. https://plato.stanford.edu/archives/spr2022/entries/mohism

[49] Hu Y, Broome M: Leadership characteristics for interprofessional collaboration in China. J Prof Nurs 36:356-363, 2020 33039070 10.1016/j.profnurs.2020.02.008

[50] Lim LC: Societal Influence, Leadership and Impact: Defining Traits of Twenty Pioneer Southeast Asian Leaders. Institute for Societal Leadership at Institutional Knowledge at Singapore Management University, Singapore 2015. https://ink.library.smu.edu.sg/cgi/viewcontent.cgi?article=1003&context=isl_research

[51] Cheng CY, Lee F: The malleability of bicultural identity integration (BII). J Cross-Cult Psychol 44:1235-1240, 2013

[52] Chan SCH, Huang X, Snape E, et al: The Janus face of paternalistic leaders. Authoritarianism, benevolence, subordinates organization-based self-esteem, and performance. J Organ Behav 34:108-128, 2012

[53] Chen CC, Farh J: Developments in understanding Chinese leadership: Paternalism and its elaborations, and alternatives, in Bond MH (ed): Handbook of Chinese Psychology. Oxford, UK, Oxford University Press, 2010, pp 599-622

[54] Farh JL, Liang J, Chou LF, et al: Paternalistic leadership in Chinese organizations: Research progress and future research directions, in Chen CC, Lee YT (eds): Leadership and Management in China: Philosophies, Theories, and Practices. London, Cambridge University Press, 2008. pp 171-205

[55] Wu M, Huang X, Li C, et al: Perceived interactional justice and trust-in-supervisor as mediators for paternalistic leadership. Manag Organ Rev 8:97-121, 2012

[56] Lu L, Zhou K, Wang Y, et al: Relationship between paternalistic leadership and employee innovation: A meta-analysis among Chinese samples. Front Psychol 13:920006, 2022 35846646 10.3389/fpsyg.2022.920006 PMC9286017

[57] Ratanjee V: Making leadership more effective in Asia. Gallup Bus J, 2013. https://news.gallup.com/businessjournal/165224/making-leadership-effective-asia.aspx

[58] Livermore D: Leading with Cultural Intelligence the Real Secret to Success (ed 2). New York, NY, American Management Association, 2015, pp 3-24

[59] Hamadeh N, Van Rompaey C, Metreau E: New World Bank country classifications by income level: 2021‐2022, World Bank blogs. July 1, 2021. https://blogs.worldbank.org/opendata/new-world-bank-country-classifications-income-level-2021-2022

[60] Dee EC, Eala MAB, Chua MLK, et al: Adolescents and young adults with cancer: Considerations from the Southeast Asian perspective. Pediatr Blood Cancer 69:e29593, 2022 35129873 10.1002/pbc.29593

[61] Eala MAB, Dee EC, Ginsburg O, et al: Financial toxicities of cancer in low- and middle-income countries: Perspectives from Southeast Asia. Cancer 128:3013-3015, 2022 35713589 10.1002/cncr.34353

[62] Harding R: Building evidence and capacity in global health palliative care. Ecancermedicalscience 16:1378, 2022 35702407 10.3332/ecancer.2022.1378 PMC9117001

[63] Allio RJ: Leaders and leadership—Many theories, but what advice is reliable? Strategy Leadersh 41:4-14, 2012

[64] Chen CC, Lee YT: Leadership and Management in China: Philosophies, Theories and Practices. Cambridge, Cambridge University Press, 2008

[65] King P, Wei Z: Chinese and western leadership models: A literature review. J Manag Res 6:1, 2014

[66] Simmonds A: Asian vs. western leadership styles, 2016. https://www.linkedin.com/pulse/asian-vs-western-leadership-styles-andrew-simmonds/[23.11.2018]

[67] Koo H, Park C: Foundation of leadership in Asia: Leader characteristics and leadership styles review and research agenda. Asia Pac J Manag 35:697-718, 2018

[68] Chiu C, Kim Y, Wan WWN: Personality: Cross-cultural perspectives, in Boyle GJ, Matthews G, Saklofske DH (eds): Personality Theories and Models. Thousand Oaks, CA, Sage, 2008. p 1

[69] Conte VA, Novello D: Assessing leadership in a Chinese company: A case study. J Manag Dev 27:1002-1016, 2008

